# A Study of One-Day Bilateral Endoscopic Tympanoplasty vs. Conventional Microscopic Tympanoplasty

**DOI:** 10.7759/cureus.77424

**Published:** 2025-01-14

**Authors:** Stuti Shukla, Sama Rizvi, Raphella Khan, Rohit Saxena, Sanjeev K Awasthi, Somya Gupta

**Affiliations:** 1 Otorhinolaryngology, School of Medical Sciences and Research, Sharda University, Greater Noida, IND; 2 Otorhinolaryngology and Surgery, Hakeem Abdul Centenary Hospital, Hamdard Institute of Medical Sciences and Research, New Delhi, IND

**Keywords:** bilateral endoscopy, chronic otitis media, conventional microscopy, tympanic perforations, tympanoplasty

## Abstract

Purpose

Chronic otitis media (COM) is generally characterized by tympanic membrane perforations. A vast range of antimicrobial as well as surgical procedures is done for the management of COM. This study aimed to assess the outcome of bilateral endoscopic tympanoplasty with that of conventional microscopic tympanoplasty.

Materials and methods

This is an interventional-prospective, comparative study. The study took place for one year, i.e., from September 2022 to August 2023. It was performed at the Department of Otorhinolaryngology, Sharda Hospital, School of Medical Sciences and Research, Sharda University (Greater Noida, UP, IND). The study included 66 participants who were divided into two groups, namely the bilateral endoscopic tympanoplasty group and the conventional microscopic tympanoplasty group. Ethical clearance was obtained before the initiation of the study.

Results

In this study, BMI was significant between bilateral endoscopic tympanoplasty and conventional microscopic tympanoplasty groups with a p-value of 0.03. The graft uptake rate postoperatively was found to be similar at 32 (97%) in the bilateral endoscopic tympanoplasty and 31 (94%) in the conventional microscopic tympanoplasty group. Air-bone gaps were decreased after surgery during follow-up months in the order of after 1 month, three months, and six months. Hearing was automatically improved after surgery, and the rate of improvement among the participants of both groups was similar.

Conclusion

The bilateral endoscopic and conventional microscopic tympanoplasty groups did not differ significantly from one another. Air-bone gaps were decreased in the duration of the follow-up period and were similar in both groups. However, it seemed easier to perform bilateral endoscopic tympanoplasty in a shorter time. Thus, bilateral endoscopy may have advancements over the conventional method.

## Introduction

It has been observed that around the world, a lot of medical visits happen due to chronic otitis media (COM), a complicated inflammatory and infectious condition. The majority of persons with this disorder undergo hearing loss or face hearing problems. It is mainly characterized by the inflammation of the tympanic membrane of the middle ear [1,2]. Chronic otitis media sequels perforation of the tympanic membrane [3].

Tympanoplasty has been a routinely performed surgical procedure in the field of otorhinolaryngology. This procedure appertains to the restoration of the hearing mechanism and removal of middle ear disease with or without tympanic membrane graft repair [4,5]. In instances of bilateral mucosal COM, the patient has to routinely wait for three months in between both surgeries. Conventionally, each eardrum was removed for grafting in two stages, which significantly increases the expense, duration, and discomfort of the procedure for the patient [6].

Bilateral tympanic membrane perforations can be closed on the same day, making it quicker and more comfortable for the patients. However, because of the potential danger of postoperative problems, conventional bilateral same-day tympanoplasty or myringoplasty has not been carried out very often [7,8]. According to a study by Rai et al., bilateral tympanoplasty type I conducted on the same day was considered a feasible therapeutic option in contemporary otology for specific populations of patients with COM [9].

According to reports, bilateral tympanoplasty performed on the same day results in iatrogenic hearing loss with a probability between 1.2% and 2.5%. As a result, otological surgeons generally advise not to perform bilateral tympanoplasty on the same day [10-12]. However, bilateral endoscopic tympanoplasty is rarely performed due to the potential risk it possesses. The dearth in the number of studies on same-day endoscopic tympanoplasty makes further studies imperative to prove its efficiency.

However, as our approach for the study is endoscopy, we sought to establish the beneficial outcomes of single-sitting bilateral endoscopic tympanoplasty compared with those of conventional microscopic tympanoplasty. Thus, the rationale of this study is to examine the outcome of single-sitting bilateral endoscopic tympanoplasty compared to conventional microscopic tympanoplasty in patients with mucosal COM in a tertiary care center.

## Materials and methods

Study design

This is an interventional-prospective, comparative study conducted at the Department of Otorhinolaryngology, Sharda Hospital, School of Medical Sciences and Research, Sharda University (Greater Noida, UP, IND). The study has been approved by the Institutional Ethics Committee of the School of Medical Sciences and Research, Sharda University (approval no. SU/SMS&R/76-A/2022/161) dated 2 August 2022. The study was conducted for one year, i.e., from September 2022 to August 2023.

Study population

The study enrolled a total of 66 patients with COM. The inclusion criteria for enrollment were patients aged 17 to 65 years, patients with either bilateral or unilateral inactive mucosal COM, and isolated conductive hearing loss. The exclusion criteria were patients with ages less than 17 years and more than 65 years, patients with inactive mucosal COM, patients with a squamosal type of disease present in ossicles, patients with sensorineural hearing loss, patients with mixed hearing loss, and patients with medical contraindications for surgery. For bilateral endoscopic tympanoplasty, 33 patients were taken into consideration whose ears were affected, while for conventional microscopic tympanoplasty, patients with unilaterally affected ears were enrolled. 

Group A included patients undergoing single-day bilateral endoscopic tympanoplasty (n=33) along with 66 ears. Type 1 tympanoplasty procedure was performed; the tympanomeatal flap was not elevated, and the edges of the perforation were freshened up under the endoscopic guide. The cartilage graft was harvested from the concha by preserving the perichondrium at one side. Using 0° and 30° endoscopes, the middle ear mucosae and ossicles were examined. A radial incision was made at the posterior-superior side of the tympanic membrane in situations when the size of the perforation made it impossible to evaluate the middle ear. The surgery was performed under local anesthesia.

Group B included patients undergoing unilateral conventional microscopic tympanoplasty (n=33). Under the guidance of the surgical microscope, the tympanomeatal flap was raised after the postauricular incision was made. Following examination of the middle ear structures and freshening of the perforation's edges, the conchal cartilage was removed, cut, and positioned utilizing the underlay technique as previously mentioned. Type 1 tympanoplasty was performed generally. The surgery was performed under general anesthesia.

Data collection

All the patients within the ambit of inclusion criteria were selected alternatively for conventional microscopic tympanoplasty and bilateral tympanoplasty in a single-day sitting. They were then followed up postoperatively at one week, four weeks, three months, and six months for further measurements of relative parameters such as air-bone gap. The data that was collected included age, gender, BMI, weight, location of perforation of the ear, and clinical manifestations like hearing loss, otorrhea, tinnitus, otalgia, and vertigo.

Statistical analysis

Analysis was performed by recording, categorizing, and computing with the help of Microsoft Excel (Microsoft Corp., Redmond, WA, USA). Data were analyzed using SPSS Statistics version 21.0 (IBM Corp., Armonk, NY, USA). Data has been presented as either mean±SD or n (%). The chi-square test was used to analyze quantitative variables. The categorical variables were compared using a t-test. An analysis of variance (ANOVA) test was used to compare the means of both groups. The p-value was considered significant at less than 0.05.

## Results

Table 1 depicts the baseline demographic characteristics of participants at baseline. The mean age of participants in the bilateral endoscopy tympanoplasty group was 36.5±9.38, and the conventional microscopic tympanoplasty group was 37.1±8.46. Similarly, BMI was 20.1±2.5 in the bilateral endoscopic group and 21.3±1.9 in the conventional microscopic group. The p-value was significant at 0.03 in terms of BMI.

**Table 1:** Baseline characteristics of participants Data are presented as either mean±SD or n (%). The p-value was considered significant at <0.05. The independent t-test was used to obtain the p-value.

Characteristics	Bilateral endoscopy tympanoplasty (n=33)	Conventional microscopic tympanoplasty (n=33)	p-value
Age (in years)	36.5±9.38	37.1±8.46	0.7
Male	19	23	-
Female	14	10	-
BMI (kg/m^2^)	20.1±2.5	21.3±1.9	0.03
Location of perforation in the ear (n=66)			
Anterior	04	03	-
Posterior	34	15	-
Central	28	15	-
Clinical manifestations (n=66)			
Hard of hearing	41 (62.1%)	23 (69.6%)	-
Otorrhea	16 (24.2%)	07 (21.2%)	-
Tinnitus	24 (36.3%)	06 (18.1%)	-
Otalgia	02 (3.03%)	00 (0%)	-
Vertigo	00 (0%)	00 (0%)	-

**Figure 1:**
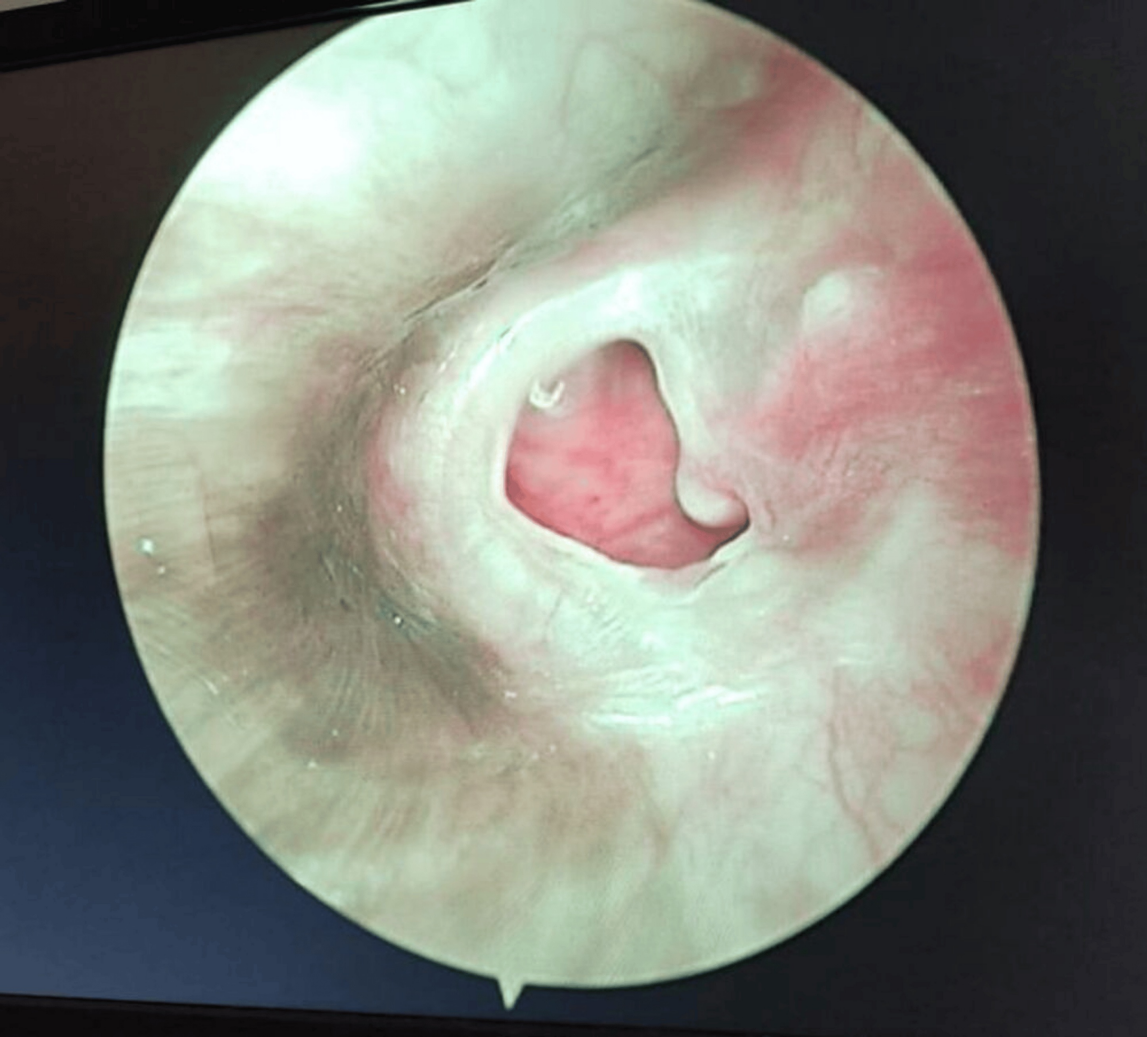
Preoperative image of the right ear of a 36-year-old female scheduled for bilateral endoscopic tympanoplasty shows perforations in the tympanic membrane

**Figure 2:**
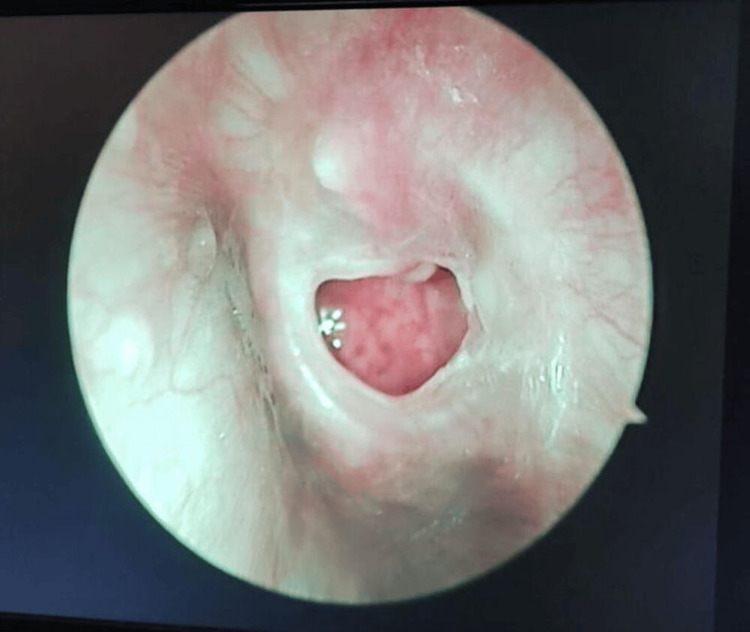
Preoperative image of the left ear of a 36-year-old female scheduled for bilateral endoscopic tympanoplasty shows perforations in the tympanic membrane

**Figure 3:**
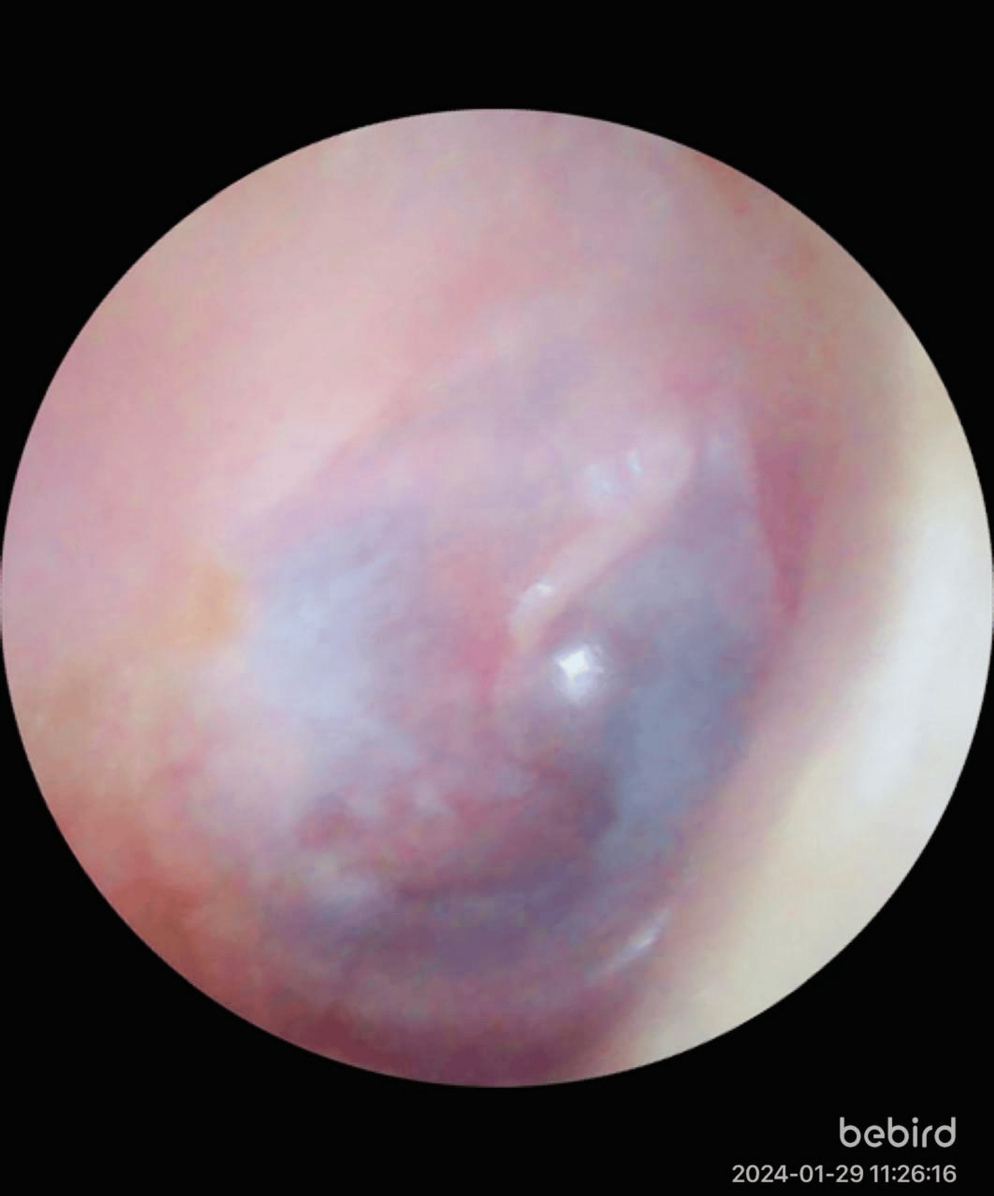
Postoperative image of the right ear of a 36-year-old female showing graft uptake after bilateral endoscopic tympanoplasty

**Figure 4:**
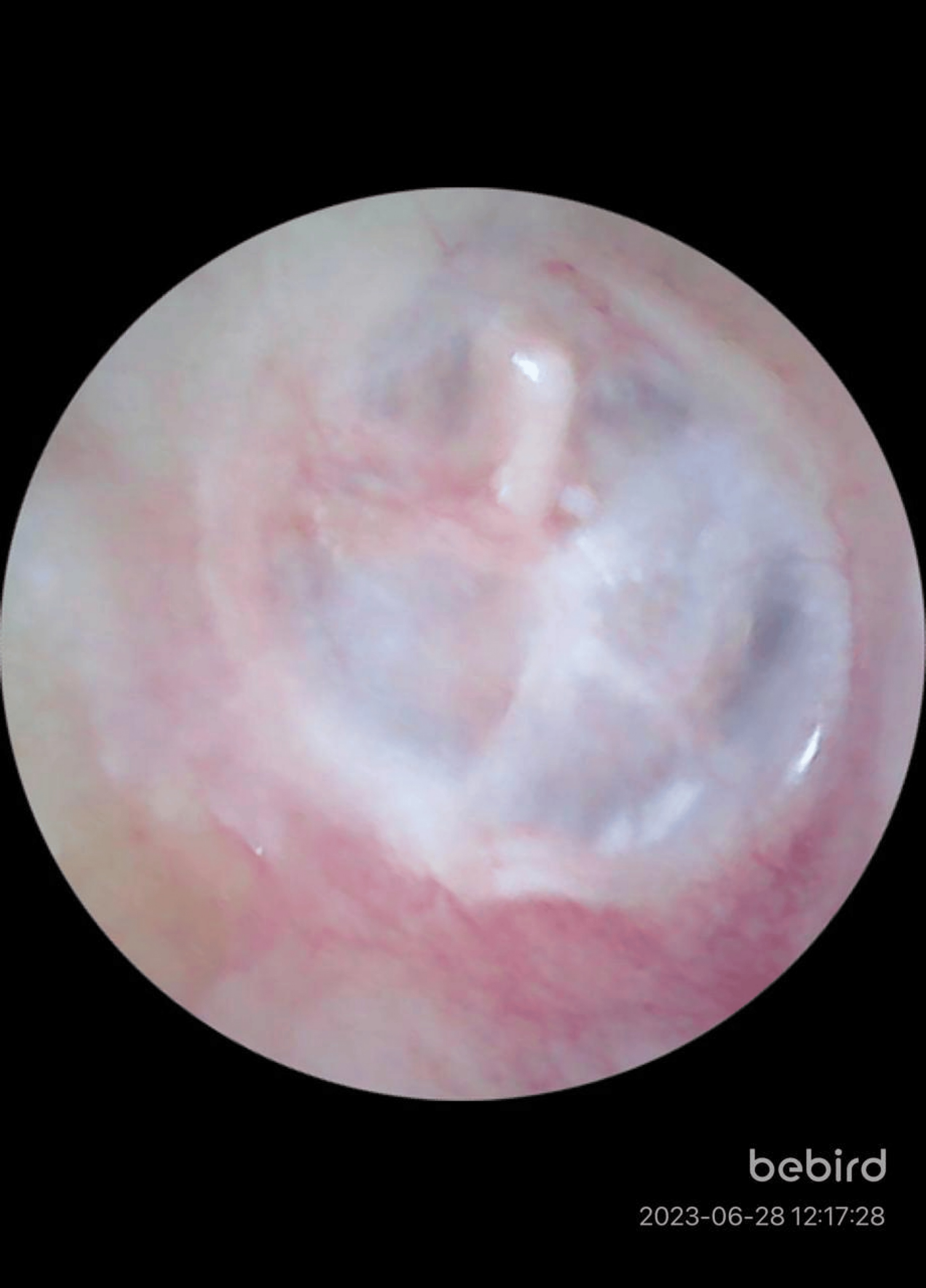
Postoperative image of the left ear of a 36-year-old female showing graft uptake after bilateral endoscopic tympanoplasty

To understand the changes that occur after tympanoplasty, some associated figures have been attached. Figures 1-2 indicate preoperative tympanoplasty of both right and left ears. Figure 3 and Figure 4 represent postoperative tympanoplasty of both the right and left ear, respectively. Table 2 represents graft uptake by the tympanic membrane after surgery. There was no statistically significant difference between both groups.

**Table 2:** Graft uptake after surgery Data are presented as number (percentage). The p-value was considered significant at <0.05. The chi-square test was used to establish a comparison between the groups.

Outcomes	Bilateral endoscopy tympanoplasty (n=66)	Conventional microscopic tympanoplasty (n=33)	p-value
Graft uptake	64 (97%)	31 (94%)	0.23
Residual perforation	02 (03%)	02 (06%)	0.23

The air-bone gap was assessed initially at the preoperative stage and then afterward in follow-up periods at one month, three months, and six months. It has been observed that air-bone gaps were declining with time, showing improvement in hearing after surgery. Table 3 presents the air-bone gap values in both groups preoperatively and postoperatively.

**Table 3:** Comparison of preoperative and postoperative air-bone gap values Data are presented as mean±SD.

Type of surgery	Preoperative air-bone gap (in dB)	Postoperative air-bone gap (in dB)		
		1 month	3 months	6 months
Bilateral endoscopic tympanoplasty	23.1±5.3	16.8±3.4	14.3±4.1	12.6±3.7
Conventional microscopic tympanoplasty	21.6±4.9	15.9±4.1	14.1±3.7	12.4±2.9

For better measurements of results and comparison, hearing gain was compared between both groups. In the bilateral endoscopy tympanoplasty group, the hearing capacity of four participants ranged between 0-5 dB, and the hearing capacity of 16 participants ranged between 10-15 dB in both ears. Similarly, in the conventional microscopic tympanoplasty group, three participants could hear between 0-5 dB, while 15 participants could hear between 10-15 dB. Table 4 shows the average hearing gain postoperatively in the participants of both groups.

**Table 4:** Average hearing gain in the respective groups Data are presented as n (%).

Hearing gain (ranges)	Bilateral endoscopy tympanoplasty (n=66)	Conventional microscopic tympanoplasty (n=33)
0-5 dB	08 (12.1%)	03 (09%)
5-10 dB	06 (09%)	06 (18.1%)
10-15 dB	32 (48.4%)	15 (45.4%)
15-20 dB	14 (21.2%)	08 (24.2%)
>20 dB	06 (09%)	01 (03%)

## Discussion

The present study was conducted to compare outcomes of bilateral endoscopic tympanoplasty with conventional microscopic tympanoplasty. It was found that BMI was significant between the groups with a p-value <0.05. In the study, most of the enrolled participants were male as compared to females. In concordance with our study, a similar study was performed earlier by Yang et al. in 2022 that also featured more male participants [13]. The study showed a similar rate of graft uptake in participants. No significant difference between graft uptake and residual perforation was observed between the groups in our study. A similar study done by Sarvya et al. in 2022 established that most of the participants had reuptake graft after a certain period [5].

Our study reported no significant differences in terms of air-bone gap values between the respective groups of bilateral endoscopic tympanoplasty and conventional microscopic tympanoplasty preoperatively as well as postoperatively after one month, three months, and six months. A similar study conducted by Daneshi et al. in 2020 concluded the same, that there was no significant difference in air-bone gaps between the procedure of bilateral endoscopic tympanoplasty and conventional microscopic tympanoplasty [1].

However, there was a decreasing difference observed in air-bone gaps postoperatively in both the procedures of bilateral endoscopic tympanoplasty and conventional microscopic tympanoplasty, respectively. A similar prospective study done by Dursun et al. in 2019 revealed postoperative air-bone gap decreased significantly with a p-value <0.001. It also concluded that bilateral same-day endoscopic tympanoplasty is a feasible surgical procedure with good anatomic and functional outcomes [14].

Equal improvements have been shown in the hearing of both groups in our study. However, the duration of bilateral endoscopic was shorter compared to conventional microscopic tympanoplasty. So, it was concluded that comparatively bilateral endoscopic tympanoplasty is more convenient than conventional microscopic tympanoplasty. Similarly, a study performed by Huang et al. in 2016 reported equal improvements in hearing capability and air-bone gaps [15]. A study conducted by Kim et al. in 2020 to compare endoscopic tympanoplasty and microscopic tympanoplasty showed that endoscopic tympanoplasty is a safer, more efficacious, and cost-effective procedure than microscopic tympanoplasty [16]. Various other studies depicted similar results, which show that patients who underwent endoscopic treatment had better outcomes [17-19].

A limitation of the study is its small sample size. Due to fewer participants enrolled in the study, the accuracy of the result may vary. Another limitation could be the follow-up period, as an extended follow-up period may result in more reliability on approaches like hearing gain postoperatively. The strength of the study is that not many studies have been performed to confirm the efficiency of bilateral endoscopic tympanoplasty over conventional microscopic tympanoplasty. Our study observed an efficient point for bilateral endoscopy, that it is easy to perform within a short period.

## Conclusions

The study observed no statistical difference between the groups of bilateral endoscopic tympanoplasty and conventional microscopic tympanoplasty in terms of graft uptake. Both procedures achieved similar outcomes, despite it being easier to perform a bilateral endoscopic tympanoplasty. As bilateral endoscopic tympanoplasty is more convenient and faster to perform than conventional microscopic tympanoplasty, there should be further investigations to support the use of the former. Bilateral endoscopic tympanoplasty is proved to be a safer procedure as it can be performed on the same day and is minimally invasive. Also, tympanoplasty for both ears can be attempted at the same time. Furthermore, the scar is prevented on the opposite side, and only a side scar is seen as the graft is harvested from one ear only.
